# High-Throughput Sequencing of Phage Display Libraries Reveals Parasitic Enrichment of Indel Mutants Caused by Amplification Bias

**DOI:** 10.3390/ijms22115513

**Published:** 2021-05-24

**Authors:** Sander Plessers, Vincent Van Deuren, Rob Lavigne, Johan Robben

**Affiliations:** 1 Department of Chemistry, KU Leuven, Celestijnenlaan 200G, B-3001 Heverlee, Belgium; sander.plessers@kuleuven.be (S.P.); vincent.vandeuren@kuleuven.be (V.V.D.); 2 Department of Biosystems, KU Leuven, Kasteelpark Arenberg 21, B-3001 Heverlee, Belgium; rob.lavigne@kuleuven.be

**Keywords:** phage display, AlkB, FTO, Illumina sequencing, Oxford nanopore sequencing, parasitic enrichment, amplification bias

## Abstract

The combination of phage display technology with high-throughput sequencing enables in-depth analysis of library diversity and selection-driven dynamics. We applied short-read sequencing of the mutagenized region on focused display libraries of two homologous nucleic acid modification eraser proteins—AlkB and FTO—biopanned against methylated DNA. This revealed enriched genotypes with small indels and concomitant doubtful amino acid motifs within the FTO library. Nanopore sequencing of the entire display vector showed additional enrichment of large deletions overlooked by region-specific sequencing, and further impacted the interpretation of the obtained amino acid motifs. We could attribute enrichment of these corrupted clones to amplification bias due to arduous FTO display slowing down host cell growth as well as phage production. This amplification bias appeared to be stronger than affinity-based target selection. Recommendations are provided for proper sequence analysis of phage display data, which can improve motive discovery in libraries of proteins that are difficult to display.

## 1. Introduction

Polypeptide molecules are displayed on the surface of filamentous bacteriophage typically by fusion to either the major (G8P) or minor (G3P) coat protein, and probed for interaction with target ligand in a process called biopanning [1]. The major power of phage display technology resides in the huge number of phage particles (>10^13^), which is amenable to selection. A large pool of variants, typically in the range of 10^7^ to 10^9^ independent clones, is redundantly represented, enabling efficient probing of the total sequence space of the library. A recent add-on to the phage display technology is the implementation of deep sequencing to investigate the library diversity. Deep sequencing (e.g., Illumina sequencing) allows for comprehensive screening of selected clones in contrast to conventional screening by ELISA and Sanger sequencing [2,3]. Indeed, since deep sequencing platforms readily provide large numbers (>10^6^) of short (100 to 400 bp) sequence reads with high accuracy [4], significantly larger fractions of the phage population can be mapped, providing more detailed insights into library complexity and enrichment. For example, deep sequencing has enhanced the understanding of various immunologic repertoires from zebrafish [5] and human heavy and light chain repertoires from isolated naive [6] and antigen-specific B-cells [7], T-cell receptors [8] and antibody display repertoires [9]. However, one limitation of high-throughput sequencing is the size of the encoded genes to be verified. Since commonly used antibody fragment formats (e.g., scFv and Fab) are usually larger than 700 bp [10], most current sequencing platforms only cover a fraction of the coding sequence (the mutagenized variable regions), meaning sequences outside the target region (constant regions, vector elements) often remain unverified.

Despite the power and robustness of the display technology, certain parameters can interfere with the selection-amplification procedure. First, since protein-displaying phage are assembled at the inner membrane of the bacterial host cells, proper translocation to the periplasmic space and folding of the coat protein fusion are crucial. Secretion difficulties may result in cellular toxicity, inefficient phage production or compromised phage infectivity [11,12,13,14]. Most often, the target protein is exported post-translationally in an unfolded state via the Sec-dependent secretion pathway. The signal recognition particle (SRP) pathway was shown to improve phage display by secreting the protein co-translationally [15,16]. Alternatively, Speck et al. explored the twin-arginine translocation (TAT)-dependent pathway for translocating the fusion protein in a folded state, allowing more efficient display of proteins requiring cytoplasmic factors, cofactors or oligomerisation for correct folding [17]. Second, phage propagation is prone to bias. Selected phagemid-bearing particles are amplified by host cell infection and rescue of the phagemid with helper phage, to serve as an input for subsequent rounds of selection. Bias may occur when certain clones amplify more efficiently than other library members and thus become enriched for reasons unrelated to target binding and selection [18,19]. Unequal phage propagation can result in the overrepresentation of so-called ‘parasitic’ sequences and, eventually, in the identification of false target-binding motifs. Many of these false-positive binders, also referred to as target-unrelated peptides or TUPs, have been reported for G8P and G3P peptide libraries [20,21,22,23]. In addition, mutations in regulatory elements in the vector, such as a ribosome binding site [24,25] or the origin of replication [26], have been shown to similarly enhance the propagation of parasitic clones.

Here, we report on amplification bias occurring in focused display libraries of two homologous nucleic acid modification eraser proteins, *E. coli* AlkB and human fat-mass and obesity-associated (FTO) protein selected for binding to methylated DNA, resulting in enrichment of display-defective, fast-growing insertion/deletion (indel) clones. We observed that high-throughput (Illumina) sequencing of the mutagenized region was not sufficient to correctly identify target-binding phenotypes since certain parasitic genotypes, especially for FTO, remained undetected. By implementing long-read (nanopore) sequencing of the entire vector, we identified unprecedented genotypes with large gene deletions and mutations outside the mutagenized region. Together, our findings could support sequence analysis and motive discovery in amplification-biased libraries.

## 2. Results and Discussion

### 2.1. AlkB and FTO Are Amenable to Phage Display

AlkB and FTO are members of the α-ketoglutarate/iron-dependent dioxygenases and are involved in the oxidative removal of a set of nucleobase modifications. AlkB is a 26 kDa *E. coli* protein involved in DNA alkylation damage repair [27], preferentially demethylating N1-methyladenosine (m1A) and N3-methylcytosine (m3C) in ssDNA [28]. FTO is a 58 kDa human protein which preferentially demethylates regulatory N6-methyladenosine (m6A) [29] and N6,2′-O-dimethyladenosine (m6A_m_) [30] in RNA. For this project, AlkB and FTO were chosen as templates for molecular evolution because of their unique base-flipping binding mechanisms and promiscuous substrate specificity. Potential is assumed for the evolution of these scaffolds for improved binding properties towards more specific nucleic acid modifications. Such evolved binders could be suited as antibody-alternatives in applications ranging from biosensing of DNA damage to nucleobase modification mapping.

**Figure 1:**
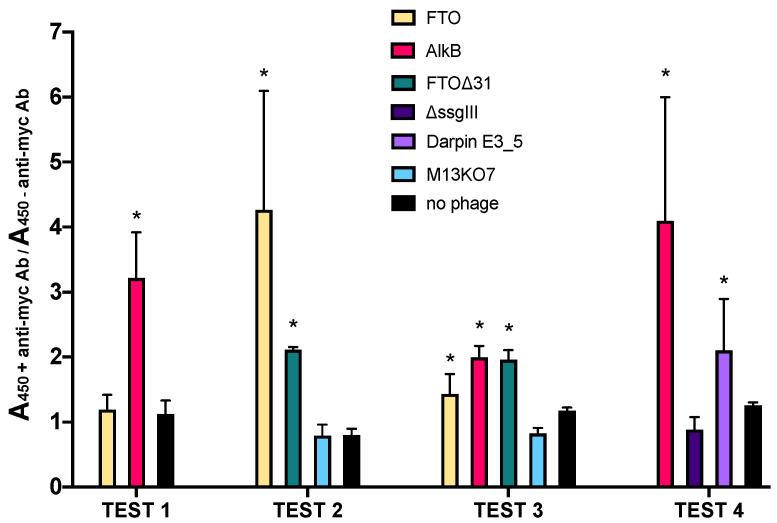
Assessment of effective display by phage ELISA. Display of target protein was measured indirectly by detection of the fused c-myc tag with anti-c-myc antibody. Bars represent absorbance ratios determined in presence (+Ab) and absence (-Ab) of immobilized anti-c-myc antibody. Since not all tests featured the same experimental variables (e.g., sample prep, age), data from different tests are shown separately. Ratios within each test are the mean of at least six technical replicates. Asterisks (*) indicate that the ratio of a particular sample within one test is significantly higher (*p*-value < 0.05) compared to the no-phage control.

Functional display requires target proteins to be transported to the periplasm, where folding occurs in a G3P membrane-anchored state, prior to incorporation into the phage. Therefore, effective display of AlkB and FTO was verified by phage ELISA using anti-c-myc antibody, recognizing the c-myc tag as part the ssG3P fusion protein in the pDST32 phagemid system (Figure S1). Absorbance values resulting from phage binding in the presence or absence of anti-c-myc were measured for different phage preparations (Figure 1). Phagemid-containing particles displaying Darpin E3_5 [15] were used as positive control. Negative controls included (i) phagemid-containing particles lacking the complete ssG3P expression cassette (pDST32-∆ssgIII), (ii) M13KO7 helper phage and (iii) solution without phage. FTO∆31, a constructed mutant lacking 31 N-terminal residues serving as human nuclear localization signal (NLS), was added to investigate potential effects of the NLS on display of FTO.

Absorbance ratios were generally around 1 for all negative control samples, meaning that no difference in absorbance was observed between the wells with and without immobilized anti-c-myc antibody. In contrast, the absorbance ratios for AlkB and FTO displaying phagemid particles were in most cases significantly higher than 1, although quite variable between different tests, indicating effective display. The lower FTO absorbance ratios compared to those of AlkB may indicate reduced incorporation of the FTO fusion protein in the phage coat. This might be attributed to a lower amount of correctly folded protein in the periplasm [13]. It is also possible that FTO is less efficiently secreted via the SRP-dependent pathway due to co-translational folding issues impairing translocation through the plasma membrane. Improved display of FTO∆31 compared to wild-type FTO indicates that the NLS may be at least partly held responsible for the less adequate display of full-length FTO.

### 2.2. High-Throughput Sequencing Reveals Enrichment of Parasitic FTO Genotypes

**Figure 2:**
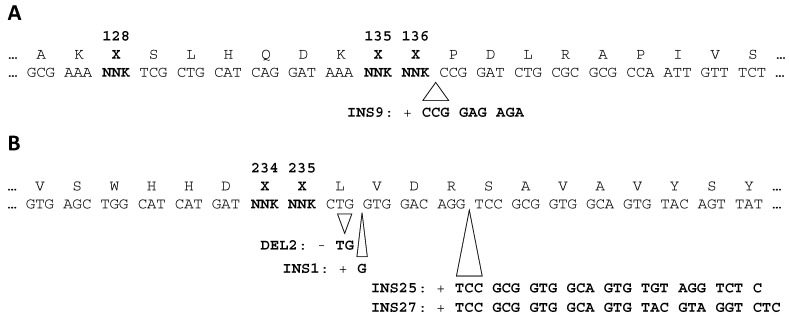
Enriched insertions (INS) and deletions (DEL) observed downstream of randomized codons (bolt) in AlkB panel (**A**) and FTO panel (**B**) coding sequences.

Randomized AlkB and FTO libraries were constructed and selected for binding of methylated ssDNA target in four panning rounds. The phagemid output after each round was analysed by Illumina sequencing of the PCR-amplified mutagenized regions. Several frequently occurring genotypes were detected (Figure 2) and the most prominent ones were further scrutinized. Different *alkB* sequence variants without insertions or deletions (further referred to as canonical sequences) as well as a specific 9 bp insertion mutation (INS9) appeared to be enriched. For *FTO*, in contrast, three short insertions (INS25, INS27, INS1) and one deletion (DEL2) occurred at high frequencies. INS25, INS1 and DEL2 are frame-shift mutations, whereas INS9 and INS27 are in-frame mutations. All indels were also found in the primary library, albeit at low frequencies, and the same mutations recurred in combination with different targeted randomized codons. Therefore, the indels presumably represent library construction artefacts. For instance, INS25, which contains an *FTO* primer-specific 25 bp duplication, can be explained as the result of mis-priming.

By comparing clone frequencies in different panning rounds in the sequencing output, opposite dynamic behaviours could be observed between AlkB and FTO phage (Table 1). In case of *alkB*, canonical clones were increased from 77% of the total paired reads before selection to 86% after four selection rounds. In contrast, unintended insertion mutants (such as INS9) gradually depleted from the library from 17% to 4%. The slow depletion of INS9 from the phage pool might be explained by reduced binding activity caused by the three-amino acid insertion close to the substrate binding site. The opposite was observed for *FTO*, where indel variants INS25, INS1 and DEL2 were substantially enriched at the expense of the canonical clones which decreased from 68% to 6%. The most frequent variant was frameshift mutant INS25, occupying more than 1/4th of the sequenced genotypes in round 4. Enrichment of INS25 mainly took place during the first selection round, showing a nearly 20-fold increase in frequency. Frameshift mutants INS1 and DEL2 enriched to 8% and 7% in round four, respectively, and showed an equal (700-fold) overall increase. In contrast, in-frame INS27, which encodes a nine-amino acid insertion *FTO* variant, did not show clear enrichment. Overall, it seems that in-frame *FTO* clones became predominated by frameshifted clones during biopanning. This indicated that loss of expression of the FTO-ssG3P fusion product rather than target binding had been driving genotypic enrichment.

Furthermore, the total number of read pairs remaining after in silico processing was more consistent for *alkB* compared to *FTO*. The number of processed *alkB* sequences is similar for each selection round (except for the naive library, due to a higher concentration of input DNA used for sequencing). In contrast, the number of processed *FTO* sequences in round 4 was reduced tenfold compared to the naive library. When verifying the different pre-processing steps (Table S1), it appeared that many reads were filtered out because they did not align with the *FTO* region targeted for sequencing. This indicates that the round 4 phagemid pool contained a significantly lower number of canonical clones and an increased number of clones partially or entirely lacking the *FTO* sequencing region. A substantial increase of deletion clones was confirmed by restriction analysis of the total phagemid DNA pool after one, two and four selection rounds (Figure S2).

### 2.3. Nanopore Sequencing Allows Detection of Genotypes Not Observed by Illumina Sequencing

**Figure 3:**
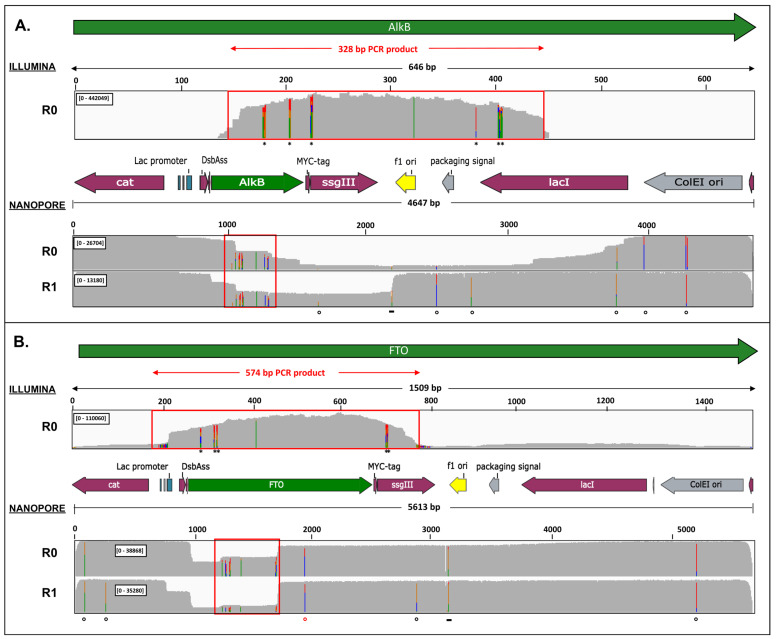
Sequence coverage of Illumina and nanopore sequence reads of the AlkB (**A**) and FTO (**B**) libraries before (R0) and after one selection round (R1). Sequence coverage (gray scale) was obtained after Illumina read processing (quality/adapter trimming, pairing) of PCR-amplified, tagmented target regions (top part of each panel). Nanopore sequence coverage (bottom part of each panel) resulted from the alignment of quality-filtered nanopore reads of ScaI-linearized phagemids to the full display vector reference sequence. Asterisks (*,**) indicate the randomized *alkB* and *FTO* target codons. Open black dots represent recurrent substitutions which are assumed to have no functional effects. The open red dot represents a frequent ochre mutation at *FTO* codon 314. A recurrent short deletion (black bar (-)) is caused by erroneous read-out of a GC-rich region. The Illumina sequencing window is marked by a red box (shown in more detail in Figure S7). Functional features of pDST32-AlkB/FTO vectors are shown as horizontal arrows.

To further investigate these deletions missed by targeted sequencing of the randomized region, we applied long-read nanopore sequencing on linearized phagemid DNA pools of the naive and round 1 libraries. Despite many local sequencing inaccuracies intrinsically associated with the sequencing technology, large *alkB* and *FTO* deletion genotypes were unequivocally detected (Figure 3). For the original AlkB phagemid library, only 12% of the aligned reads contained the phage origin of replication and 13% contained the complete *alkB-ssgIII* sequence. Hence, it appears that the original phagemid library was heavily contaminated with deletion artefacts (~87%). After one round, including a phage rescue step, the fraction of reads with the phage origin of replication increased from 12% to 95%, but still, 63% of these missed the complete *alkB-ssgIII* sequence.

In the FTO naive library, low read coverage was mainly observed for the first half of FTO coding sequence, with at least 40% of the aligned reads containing an *FTO*-specific deletion. These deletions were artefacts generated during library construction since their position and length match with the randomized megaprimer fragment used for mutagenesis and cloning. In the round 1 library, the number of *FTO*-deletion mutants further increased to 85%, while deletions of the *lac* promoter region appeared in 38% of the reads. The INS25 and INS27 mutants were detected in the *FTO*-containing nanopore reads, while variants INS1 and DEL2 could not be sorted out with certainty.

### 2.4. Amino Acid Patterns Deduced from Enriched Genotypes Can Be False Positives

Despite the above-mentioned shortcomings, we attempted to extract affinity-enriched binding motifs from DNA sequence-deduced amino acids at the randomized positions. For both proteins, the search was limited to Illumina read pairs covering all randomized positions. This automatically excluded most of the large deletion clones observed in the nanopore reads. Since suppression of frameshift mutations by ribosomal slippage cannot be excluded [31,32], clones with small indels up- and downstream of the mutagenized region were initially not removed from the dataset. Motifs represented by the sequence of the randomized amino acid residues were sorted according to frequency, and top scoring patterns were compared (Table S2).

**Figure 4:**
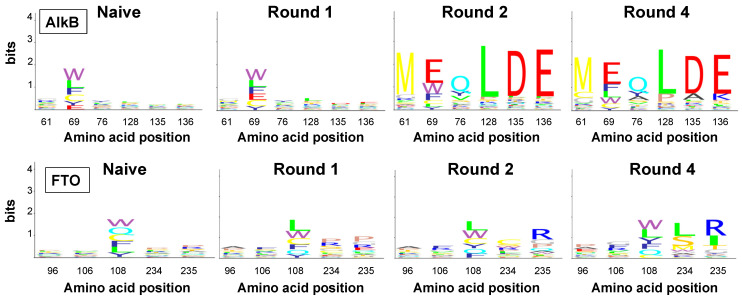
Sequence logos of canonical AlkB and FTO motifs at the six and five randomized positions, respectively, as deduced from Illumina reads. *AlkB* codon 69 and FTO codon 108 were TDK-randomized (encoding only five residues ((C, F, L, W, Y) and the amber stop codon, which is represented as Gln (Q) as it is efficiently suppressed in display strain TG1). All other triplets were NNK-randomized. Sequence logos were generated with Matlab.

For AlkB, specific amino acid combinations were revealed, in particular MWYxxx and M*Qxxx (* represents a Gln translated from an amber stop codon suppressed in TG1 [33]) at positions 61, 69 and 76; and xxxPAK and xxxLDE at positions 128, 135 and 136 (Figure 4). The most prominent pattern, M*QLDE, amounted to 28% of the paired reads already after round 2. The other patterns, including that of the wild-type AlkB (MWYLDE), showed slower enrichment, reaching only 3 to 5% in round 4. Prevalence due to compositional bias in the naive library and genetic bottleneck effects can be excluded since sequence hits matching the enriched patterns were rare in the naive library. Notably, all enriched motifs were encoded by one dominating genotype (Table S3A). A plausible explanation could be the limited primary library size and sampling of the theoretical sequence space. Therefore, the observed AlkB patterns potentially result from phenotypic selection for genuine target binding, as corroborated by the co-enrichment of wild-type AlkB. On the other hand, selective advantages caused by premature stop codons or other expression-related effects cannot be excluded.

In case of FTO, Illumina paired reads yielded specific amino acid enrichment at position 108, 234 and 235 (Figure 4). At position 108, Trp and Leu were dominating after round 4 (25% and 23%, respectively). Additionally, wild-type Tyr increased from lowest frequency in the naive library (12%) to the third most frequent amino acid in round 4 (17%). At positions 234 and 235, amino acid duplets xxxLR and xxxSI were significantly enriched from 1.32% and 0.06% in the naive library to 34% and 26% in round 4, respectively. The most frequently observed five-amino acids patterns in canonical clones were AFLLR and EGWLR, occurring at frequencies of 4.2% and 3.8% of the total read pairs, respectively (Table S2). While these clones were represented by different genotypes in the first selection round, genotypic diversity substantially decreased upon further selection (Table S3B). It is noteworthy that the AFLxx triplet motif was generally found among all indel variants as well, in combination with a variant-specific amino acid duplet (INS25: AFLVP, INS27: AFLPK, INS1: AFLSP, DEL2: AFLIS) (Table S2).

Especially, duplets xxxSP and xxxIS were extremely enriched for INS1 and DEL2, respectively, to almost 100% after round 4. These data, corroborated by the enrichment of large deletions in the nanopore sequencing output, strongly indicate that the observed AFLxx motifs were caused by indels compromising FTO display. Zhang et al. showed that mutations R96A and Y106F (positions 1 and 2 in AFLxx) reduce the FTO binding affinity for m6A-containing ssDNA [34], reinforcing that the Illumina sequence-deduced FTO motifs were not m6A affinity-enriched, but false positives.

### 2.5. Parasitic Enrichment of FTO Variants Is Enhanced by Amplification Bias

**Figure 5:**
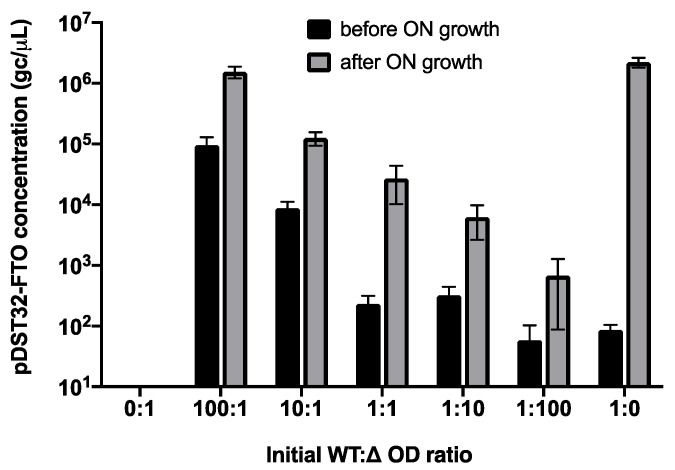
Evolution of pDST32-FTO phagemid copy numbers in mixed cultures of competing TG1/pDST32-FTO (WT) and TG1/pDST32-∆ssgIII (∆) cells. Medium was inoculated with different amounts of cells in the corresponding ratios (1 amount equalled OD = 0.001) and grown overnight at 37 °C. pDST32-FTO phagemid concentrations (gc/µL) were determined by qPCR using *FTO*-specific primers (Table S4) before and after overnight growth at 37 °C. A 10-fold dilution series of pDST32-FTO was used as standard. Data are the means of at least three independent replicates.

Since parasitic enrichment was obvious for the FTO phage library, wild-type FTO phage properties were further investigated. First, FTO phagemid amplification was monitored in mixed cultures of TG1 cells transformed with either pDST32-FTO producing displayable wild-type FTO, or with pDST32-∆ssgIII lacking the G3P-coding sequence and devoid of displayable products. The two cell types were mixed in different OD ratios and grown overnight in the absence of coat protein inducer IPTG. pDST32-FTO phagemids were PCR-quantified before and after overnight growth (Figure 5).

In pure TG1/pDST32-FTO cultures (condition 1:0), the pDST32-FTO phagemid concentration increased 10,000-fold overnight. However, when both cell types in equivalent amounts (1:1 ratio) were grown overnight, amplification of pDST32-FTO was significantly lower (100-fold increase) and reduced even further at increasing pDST32-∆ssgIII/FTO cell ratios (only tenfold increase in case of a 1:100 ratio). Alternatively, cultures containing a 100-fold excess of TG1/pDST32-FTO (100:1) showed only tenfold amplification of pDST32-FTO, which is much lower than expected. This indicates that cell growth of TG1/pDST32-FTO strongly lags behind that of the *FTO-ssgIII* gene-missing variant. This was confirmed by a parallel experiment in which both TG1 cell types were grown in separate cultures, showing a 20 min increased doubling time for TG1/pDST32-FTO (Figure S3). Since the expression of FTO-ssG3P was not induced, FTO growth issues are likely replication-related and affected by the human FTO DNA sequence rather than the protein expression product. However, leaky expression of the potentially toxic FTO fusion protein might not be excluded.

**Figure 6:**
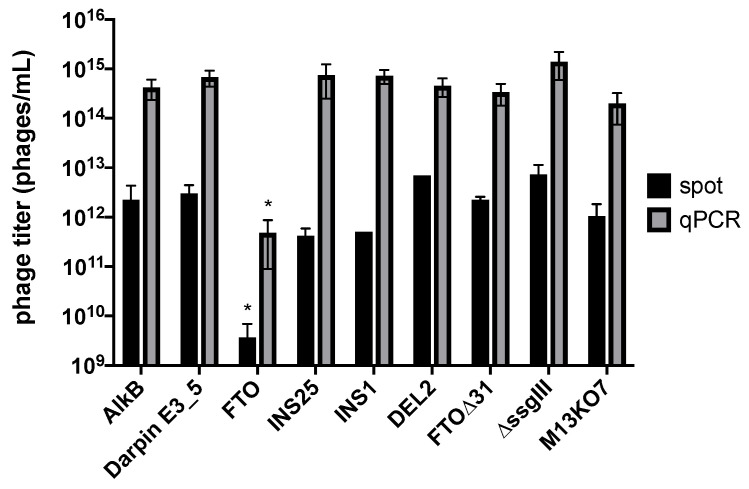
Effects of display on phage production as measured by spot titration and qPCR. FTO phage titers were compared with those of AlkB and Darpin E3_5 [15] display phages, FTO indel variants (INS25, INS1, DEL2, FTO∆31), and phagemid-containing particles lacking the complete *FTO-ssgIII* fusion gene (∆ssgIII), and M13KO7 helper phage. Data are the means of at least two biological and three technical replicates (except spot titer of INS1 and DEL2). For both methods, the FTO phage titer is ~10^3^ times lower than all other titers (indicated by *; *p* < 0.05).

Second, phage production of FTO-displaying phagemid-containing particles was investigated using qPCR and spot titration (Figure 6). While qPCR quantifies the absolute number of phage particles (or, more precisely, vector copies), spot titration determines the number of infective phage particles as it requires a TG1 infection step for titer calculation (see Section 3.2. “Materials and Methods”). The infectivity of phage particles (represented by the ratio of infective titer over the absolute titer) was relatively consistent (~0.5%) among the different phage samples. This was expected since all phage variants rely on helper phage G3P for infection. Looking at phage production, the titer of FTO-displaying particles appeared to be about three orders of magnitude less compared to the titers of all other tested phage particles. Although full-length FTO appeared displayable on phage (Figure 1), the observed reduction in FTO phage production might be caused by expression issues of the full-length FTO fusion protein, either during translocation or in the periplasm. Due to the high number of cysteine residues (14), FTO is prone to disulphide crosslinking, which might covalently trap a misfolded structure or cause periplasmic aggregation prior to phage assembly. FTO∆31, in contrast, shows normal phage titers, pointing to a secretion problem caused by the NLS of FTO. Out-of-frame, prematurely terminating FTO variants (INS25, INS1 and DEL2) show normal phage numbers, which supports this hypothesis.

Reduced amplification of full-length FTO-displaying phage particles, as a result of both reduced host cell growth and reduced phage production, can explain the false-positive biopanning results. In mixed cultures, out-of-frame or *FTO* truncation mutants amplify more efficiently by avoiding productive FTO-G3P expression (Figure 5). As a result, genuine affinity-selected FTO clones risk being outcompeted during host cell growth and phage rescue by parasitic clones which enrich independent of target binding.

## 3. Materials and Methods

### 3.1. AlkB and FTO Phage Production

AlkB and full-length FTO wild-type (WT) coding sequences (Figure S4) were cloned into phagemid pDST32 [15] (a derivative of vector pMorph7 [35] encoding Darpin E3_5 [36] and the DsbA signal sequence (DsbAss)) as a fusion with supershort G3P (ssG3P), i.e., G3P lacking the N1 and N2 domains (cloning details provided in Supplementary Materials). *E. coli* strain TG1 (K12, *Δ(lac-pro), supE, thi, hsdD5/F′traD36, proA+B+, lacIq, lacZΔM15*) (Agilent) was transformed with phagemid DNA, grown at 37 °C to OD_600_ = 0.5 and superinfected with 10^9^ plaque-forming units (pfu) M13KO7 helper phage for 30 min. Superinfected cells were centrifuged for 10 min to remove residual helper phage. AlkB and FTO display were initiated by overnight growth, respectively, at 30 °C and 18 °C in the presence of 100 µM and 10 µM IPTG. Released phage particles were harvested and purified by two 20% PEG/2.5 M NaCl precipitation steps. Residual bacterial cells were removed with a 0.22 µm membrane filter (Sartorius, Göttingen, Germany). Purified phage particles were resuspended in 1 mL TBS (AlkB) or PBS (FTO).

### 3.2. Phage Titers

Infective phagemid-containing phage particles and M13KO7 helper phage were quantified by spot titration of a tenfold dilution series of regular phage preparations. Total phage titers were determined by qPCR, as described by Peng et al. [37]. Therefore, 1000-fold diluted phage particles were pre-treated with DpnI for 10 min to remove residual cellular DNA. After incubation at 100 °C for 15 min, phage ssDNA was quantified by 40 cycles of qPCR (three biological and six technical replicates) in the presence of pDST32- or M13KO7-specific primers (Table S4) and SYBR Green 2× Master Mix (ThermoFisher Scientific, Waltham, MA, USA) in a QuantStudio3 real-time PCR-machine (ThermoFisher Scientific). A tenfold dilution series of pDST32-FTO (5613 bp) and M13KO7 dsDNA (8669 bp), ranging from 1 fg/μL to 10^7^ fg/μL, was used as standard for the calibration curve of pDST32 and M13KO7 samples, respectively. The phage standard DNA concentration was converted to genome copies per microliter (gc/μL) according to the formula of Peng et al. [37].

### 3.3. Anti-C-Myc Phage ELISA

An anti-c-myc phage ELISA was carried out on various phagemid-containing particles to estimate the efficiency of protein display. First, biotinylated anti-c-myc antibody (Novus Biologicals, Centennial, CO, USA) in 1× TBST (TBS with 0.1% Tween-20) was coated on prewashed neutravidin 96-well plate (ThermoFisher Scientific) by adding 100 µL of 1/10,000 anti-c-myc Ab TBST solution. In parallel, a blanc TBST solution without antibody was used as control. The plate was washed three times with TBST to remove unbound antibodies, after which 10^9^ to 10^11^ phage particles were added. After washing three times, captured phage particles were detected by adding a 1/5000 solution of mouse anti-M13 antibody horseradish peroxidase conjugate (SinoBiological, Wayne, PA, USA) in 1× TBST. After five wash steps, 3,3′,5,5′-tetramethylbenzidine (TMB) was added and the staining reaction stopped by 1N HCl after defined time periods. The absorbance was measured on TECAN Safire 2 microplate reader (ThermoFisher Scientific). All immobilization steps were performed at a gentle shaking speed for 1 h at room temperature. All ELISA measurements were performed in triplicate.

### 3.4. Library Generation and Biopanning

Focused six amino acid residue-randomized AlkB protein libraries (M61, W69, Y76, L128, D135, E136) and five amino acid residue-randomized FTO libraries (R96, Y106, Y108, E234, N235) were generated by Golden Gate cloning (New England Biolabs (NEB), Ipswich, MA, USA) (Figure S5) using two degenerated primers (Table S4). These residues were chosen based on their position in the nucleobase-binding pocket and their critical role in nucleobase and modification binding [38]. The primary library size estimated from the number of independent clones after transformation was 5.0 × 10^5^ and 6 × 10^6^, respectively. AlkB and FTO phage libraries were selected for binding a target oligonucleotide containing a single N1-methyladenine (m1A) or N6-methyladenine (m6A), respectively. The binding interaction was performed by mixing 10^10^ to 10^11^ phage particles with 50 µL catalysis-inhibiting binding buffer (1× TBST, 100 µM MnCl_2_ and α-ketoglutarate for AlkB; 1× PBST, 1 mM MnCl_2_ and N-oxalylglycine [39] for FTO) and incubation with 4 pmol (AlkB) or 2 pmol (FTO) biotinylated target ssDNA at 37 °C for 30 min. From the second selection round onwards, unmodified competitor oligonucleotides were added to reduce non-specific DNA binding. For phage capture, hydrophilic streptavidin-coated paramagnetic beads (NEB) were pre-incubated for 1 h with blocking buffer (1× TBST/PBST and 5% BSA), followed by four sequential wash steps in 1 mL TBST/PBST, gentle rotation, and separation by magnetic capture to facilitate removal of the supernatant. Phage suspensions were added to the equilibrated beads and were slowly rotated for 30 min at room temperature to capture m1A- or m6A-bound phage. After magnetic immobilization, non-binders were removed by 10 consecutive washing steps. The remaining phage were eluted by 100 mM triethylamine (pH = 11) for 5 min and the eluate was neutralized with 1 M Tris-HCl (pH = 7.4). Eluted phage particles (generally 10^4^–10^7^) were amplified by infecting exponentially growing TG1 cells and rescued by helper phage, as described above. An aliquot of the cell suspension was used for phagemid DNA preparation for sequencing.

### 3.5. Illumina Sequencing

Using phagemid DNA as a template, variable target regions were amplified by six cycles of PCR using gene-specific primers (Table S4), resulting in an amplicon of either 328 bp (*alkB*) or 574 bp (*FTO*). Purified PCR amplicons were randomly cleaved and tagged (“tagmented”) by transposase, generating 250 to 500 bp fragments using the Nextera Flex Prep kit (Illumina, San Diego, CA, USA). Tagmented fragments were size-selected and adapter-ligated (Nextera DNA CD Indexes). The generated DNA libraries were sequenced on the lllumina MiniSeq platform using the Miniseq Mid output kit (300 cycles) to yield 2 × 150 bp paired-end reads. Raw sequence data (FASTQ files) were adapter- and quality-trimmed using Trim Galore [40] and the FASTX-Toolkit [41]. Reads were filtered to contain target region-specific sequences (Figure S6), paired and aligned with the wild-type AlkB or FTO sequence using BBMap [42] (Figure S7). Sequence variants were detected using the CLC genomic software (Qiagen, Hilden, Germany) and IGV (Broad Institute), filtered and trimmed with Cutadapt [43] for pattern analysis. The resulting FASTQ files were further analysed using in-house Matlab scripts. DNA sequences were translated and the specific amino acids at the randomized positions were combined into a six- or five-residue amino acid pattern for AlkB and FTO, respectively (Figure S8).

### 3.6. Oxford Nanopore Sequencing

Phagemid DNA of the naive library (R0) and the first selection round (R1) was linearized by ScaI fast digestion for 30 min at 37 °C. Linearized phagemids were purified using the PCR-purification kit (ThermoFisher Scientific) and 1 µg was used for nanopore sequencing. Phagemid DNA was barcoded, adapter-ligated and loaded onto FLO-FLG001 type flow cells following the manufacturers protocol (Oxford Nanopore Technologies, Oxford, UK). Nanopore sequencing was performed on a MinION device for 48 h. Raw sequence data (FASTQ files) were quality-filtered (Q > 10) using NanoFilt [44] (Figure S9), aligned with the wild-type pDST32-AlkB/FTO vector sequence using NGMLR [45] and visualized using IGV (Figure 3). The data analysis for Figures S10 and S11 was performed using the NanoPack toolkit [44].

## 4. Conclusions

Comparative analysis of the phage display output has shown that AlkB yielded potential target binders, whereas its human homolog, FTO, suffered substantially from parasitic target-independent clone enrichment. This enrichment could be pinpointed to reduced host cell growth and phage production most likely due to impairment of protein secretion by NLS-containing FTO. Clones carrying deletions precluding FTO expression apparently propagated faster than could be compensated by the affinity selection imposed during biopanning. While parasitic enrichment is a known issue in phage display [20,21,22,23,24,25,26], we nevertheless attempted to identify genuine target binders by monitoring the phage populations utilizing high-throughput sequencing. Focused Illumina sequencing of the randomized regions revealed potential amino acid motifs, but in the case of FTO, it is mostly associated with small indels. In addition, nanopore sequencing of the entire vector revealed larger deletions, in large part unintendedly created during library construction. Illumina-deduced FTO motifs, also those without indels in the short reads, should therefore be interpreted with caution. These results highlight that high-throughput sequencing should not be restricted to the mutagenized region of the library, especially when display of the target protein appears difficult [13,14] and parasitic amplification bias is a potential risk. Although motifs might be discovered by nanopore sequencing in specific cases [46], we could not match Illumina and nanopore reads of individual clones with certainty due to the short read-lengths generated by the Illumina method on the one hand, and the less accurate local base calling of the nanopore method on the other hand. Long-read sequencing methods with improved accuracy, such as PacBio circular consensus sequencing (CCS) [47], could aid motif analysis, as recently applied to scFv phage libraries [48]. Alternatively, negative effects of amplification bias might be partly circumvented by sequencing of DNA directly isolated from eluted phage [49] instead of phagemid DNA prepared after phage infection and host cell growth. Our results also highlight the importance of high-quality library construction, as parasitic sequences mainly originated from artefacts. Improved mutagenesis strategies with higher efficiency and minimal hands-on time, such as Darwin assembly [50], for instance, might be a valuable alternative. Before engaging in panning, integral target protein expression on phage particles should be measured, e.g., by western blotting, as well as functional ligand binding by ELISA, which could warn for deletion selection issues. Finally, when displaying cumbersome target proteins, we recommend minimizing the number of amplification steps to slow down the enrichment of perturbing parasitic genotypes, thus facilitating the identification of true binding patterns.

## Figures and Tables

**Table 1:** Frequency evolution of *alkB* and *FTO* canonical (CAN) and indel (INS, DEL) clones during panning as derived from the Illumina sequencing output.

	*alkB*			*FTO*					
Library	Total Paired Reads	CAN (%)	INS9 (%)	Total Paired Reads	CAN (%)	INS25 (%)	INS27 (%)	INS1 (%)	DEL2 (%)
Naive	761,541	77.09	17.22	115,191	67.69	1.06	0.14	0.01	0
Round 1	151,953	82.26	14.14	106,962	28.59	19.88	0.61	0.76	0.06
Round 2	140,577	86.26	3.71	64,156	16.7	25.24	0.71	2.6	1.67
Round 4	128,364	85.79	4.28	9453	5.74	28.49	0.51	8.05	7.39

The first *alkB* and *FTO* column represents the total number of paired reads which remained after raw read processing and removal of non-specific reads. The subsequent columns show the fractions (%) of canonical (CAN) and indel clones in each selection round.

## Data Availability

No supporting data other than the ones in the Supplementary Material have been reported.

## Supplementary Materials

Supplementary Materials can be found at https://www.mdpi.com/article/10.3390/ijms22115513/s1.
