# Supplementary material for: High-Throughput Sequencing of Phage Display Libraries Reveals Parasitic Enrichment of Indel Mutants Caused by Amplification Bias
Source: Int J Mol Sci. 2021 May 24;22(11):5513. doi: 10.3390/ijms22115513 (PMC8197208; doi:10.3390/ijms22115513)
Supplement: Supplementary file 1 [file ijms-22-05513-s001.zip › ijms-1223945-supplementary.pdf]

Supporting Information for

High-Throughput Sequencing of Phage Display Libraries Reveals Parasitic Enrichment of Indel Mutants Caused by Amplification Bias

Sander Plessers [1], Vincent Van Deuren [1], Rob Lavigne [2] and Johan Robben [1,*]

Table of Content

Figure S1. Schematic representation of the *alkB* and *FTO* fusion constructs in pDST32 used for phage display 2

Figure S2. Restriction analysis of naive and selected FTO phagemid pools. 3

Figure S3. Doubling time of liquid-cultured TG1 cells bearing pDST32-FTO or pDST32-ΔssgIII phagemids 4

Figure S4. Cloned coding sequence of *E. coli* AlkB and human FTO 5

Figure S5. Construction schemes of AlkB and FTO libraries........ 6-7

Figure S6. Detailed overview of the targeted regions and randomized codons in *alkB* and *FTO* 8

Figure S7. Sequence coverage of the *alkB* and *FTO* gene after deep sequencing........ 9

Figure S8. Evolution of amino acids at randomized positions in AlkB and FTO during biopanning........ 10

Figure S9. NanoPlot bivariate plot of linearized pDST32-FTO nanopore read lengths against average read quality........ 11

Figure S10. NanoPlot histogram of raw nanopore read lengths of AlkB phagemid libraries 12

Figure S11. NanoPlot histogram of raw nanopore read lengths of FTO phagemid libraries . 13

Table S1. Absolute numbers of sequence reads and read pairs in consecutive steps of FASTQ processing. 14

Table S2. Frequency evolution of most prominent AlkB and FTO amino acid patterns deduced from deep sequencing reads 15

Table S3. Observed *alkB* and *FTO* genotype frequencies and diversities of enriched canonical phenotypes. 17

Table S4. Oligonucleotides used in this study........ 19

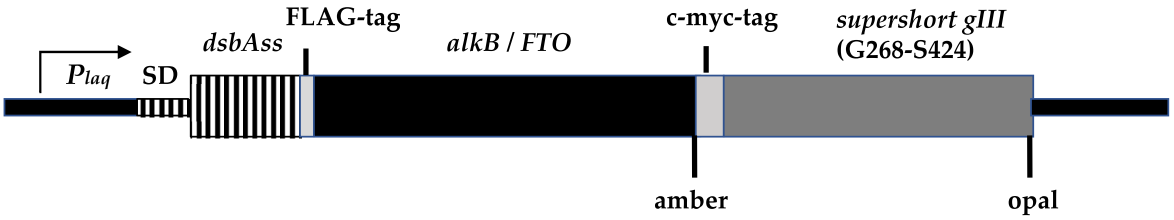

Figure S1. Schematic representation of the *alkB* and *FTO* fusion constructs in pDST32 used for phage display.

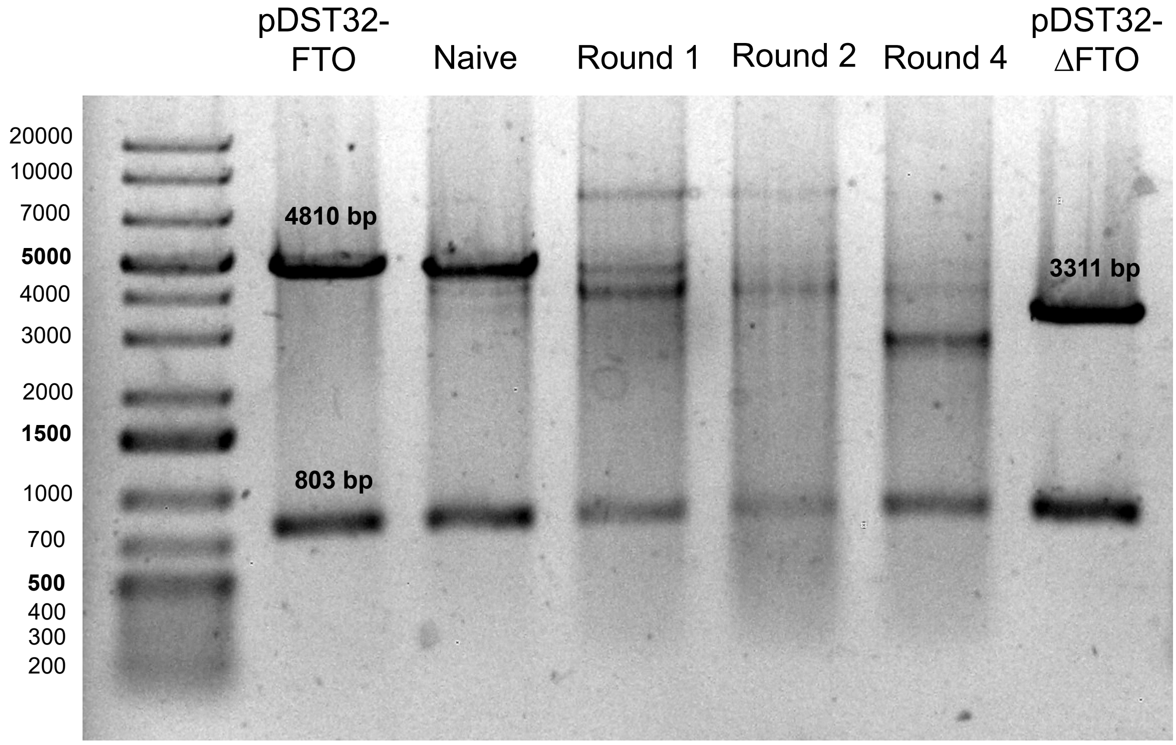

Figure S2. Restriction analysis of naive and selected FTO phagemid pools. Phagemid DNA isolated from library-transfected TG1 cell cultures (naive and round 1, 2 and 4 libraries) were cut by ScaI and NheI double-digestion. This generates an 803 bp fragment containing the origin of replication and part of the *cat* resistance marker, and an expected 4810 bp fragment containing the *FTO-ssgIII* expression module. Plasmids pDST32-FTO and pDST32-ΔFTO were used as size controls.

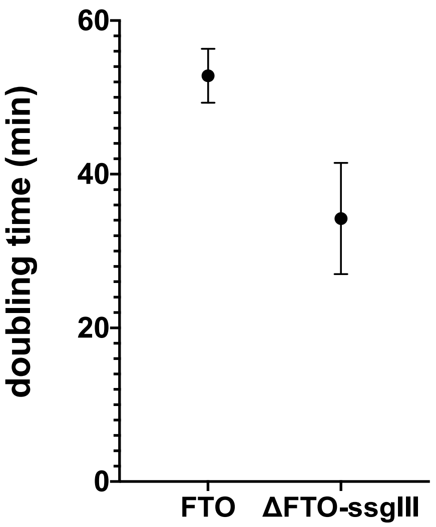

Figure S3. Doubling time of liquid-cultured TG1 cells bearing pDST32-FTO or pDST32-ΔssgIII phagemids. The OD of each cell culture was monitored for 10 hours and the doubling time was determined by plotting ln(OD) in function of time and calculating the slope of the linear part of the curve. Data are the means of three biological and three technical replicates.

AlkB

atg ttg gat ctg ttt gcc gat gct gaa ccg tgg caa gag cca ctg gcg gct ggt gcg gta
att tta cgg cgt ttt gct ttt aac gct gcg gag caa ctg atc cgc gat att aat gac gtt
gcc agc cag tcg ccg ttt cgc cag atg gtc acc ccc ggg gga tat acc atg Tcg gtg gcg
atg acc aac tgt ggg cat ctg ggc tgg acg acc cat cgg caa ggt tat ctc tat tcg ccc
att gat ccg caa aca aat aaa ccg tgg ccc gcc atg cca cag agt ttt cat aat tta tgt
caa cgt gcg gct acg gcg gcg ggc tat cca gat ttc cag cca gat gct tgt ctt atc aac
cgc tac gct cct ggc gcg aaa ctg tcg ctg cat cag gat aaa gac gaa ccg gat ctg cgc
gcg cca att gtt tct gtt tct ctg ggc tta ccc gcg att ttt caa ttt ggc ggc ctg aaa
cga aat gat ccg ctc aaa cgt ttg ttg ttg gaa cat ggc gat gtg gtg gta tgg ggc ggt
gaa tcg cgg ctg ttt tat cac ggt att caa ccg ttg aaa gcg ggg ttt cat cca ctc acc
atc gac tgc cgc tac aac ctg aca ttc cgt cag gca ggt aaa aaa gaa taa

FTO

atg ggg aag cgc acc ccg act gcc gag gaa cga gag cgc gaa gct aag aaa ctg agg ctt
ctt gaa gag ctt gaa gac act tgg ctc cct tat ctg acc ccc aaa gat gat gaa ttt tat
cag cag tgg cag ctg aaa tat cct aaa cta att ctc cga gaa gcc agc agt gta tct gag
gag ctc cat aaa gag gtt caa gaa gcc ttt ctc aca ctg cac aag cat ggc tgc tta ttt
cgg gac ctg gtt cga atc caa ggc aaa gac ctg ctc act ccg gta tct cgc atc ctc att
ggt aat cca ggc tgc acc tac aag tac ctg aac acc agg ctc ttt acg gta ccc tgg cca
gtg aaa ggg tct aat ata aaa cac acc gag gct gaa ata gcc gct gct tgt gag acc ttc
ctc aag ctc aat gac tac ctg cag ata gaa acc atc cag gct ttg gaa gaa ctt gct gcc
aaa gag aag gct aat gag gat gct gtg cca ttg tgt atg tct gca gat ttc ccc agg gtt
ggg atg ggt tca tcc tac aac gga caa gat gaa gtc gac att aag agc aga gca gca tac
aac gta acg ttg ctg aat ttc atg gac cct cag aaa atg cca tac ctg aaa gag gaa cct
tat ttt ggc atg ggg aaa atg gca gtg agc tgg cat cat gat gaa aat ctg gtg gac agg
tcc gcg gtg gca gtg tac agt tat agc tgt gaa ggc cct gaa gag gaa agt gag gat gac
tct cat ctc gaa ggc cgt gat cct gat att tgg cat gtt ggt ttt aag atc tca tgg gac
ata gag aca cct ggt ttg gcg ata ccc ctt cac caa gga gac tgc tat ttc atg ctt gat
gat ctc aat gcc acc cac caa cac tgt gtg ttg gcc ggt tca caa cct cgg ttt agt tcc
acc cac cga gtt gca gag tgc tca acc ggt aca ttg gat tat att tta caa cgc tgt cag
ttg gct ctg cag aat gtc tgt gac gat gtg gac aat gat gat gtc tct ttg aaa tcc ttt
gag cct gca gtt ttg aaa caa gga gaa gaa att cat aat gag gtc gag ttt gag tgg ctg
agg cag ttt tgg ttt caa ggc aat cga tac aga aag tgc act gac tgg tgg tgt caa ccg
atg gct caa ctg gaa gca ctg tgg aag aag atg gag ggt gtg aca aat gct gtg ctt cat
gaa gtt aaa aga gag ggg ctc ccc gtg gaa caa agg aat gaa atc ttg act gcc atc ctt
gcc tcg ctc act gca cgc cag aac ctg agg aga gaa tgg cat gcc agg tgc cag tca cga
att gcc cga aca tta cct gct gat cag aag cca gaa tgt cgg cca tac tgg gaa aag gat
gat gct tcg atg cct ctg ccg ttt gac ctc aca gac atc gtt tca gaa ctc aga ggt cag
gtt cga atc

Figure S4. Cloned coding sequence of *E. coli* AlkB and human FTO. Three extra guanines (underlined) were added to the sequence for cloning purposes, resulting in an extra glycine in the FTO translation product. The N-terminal FTO nuclear localization signal is shown in italics.

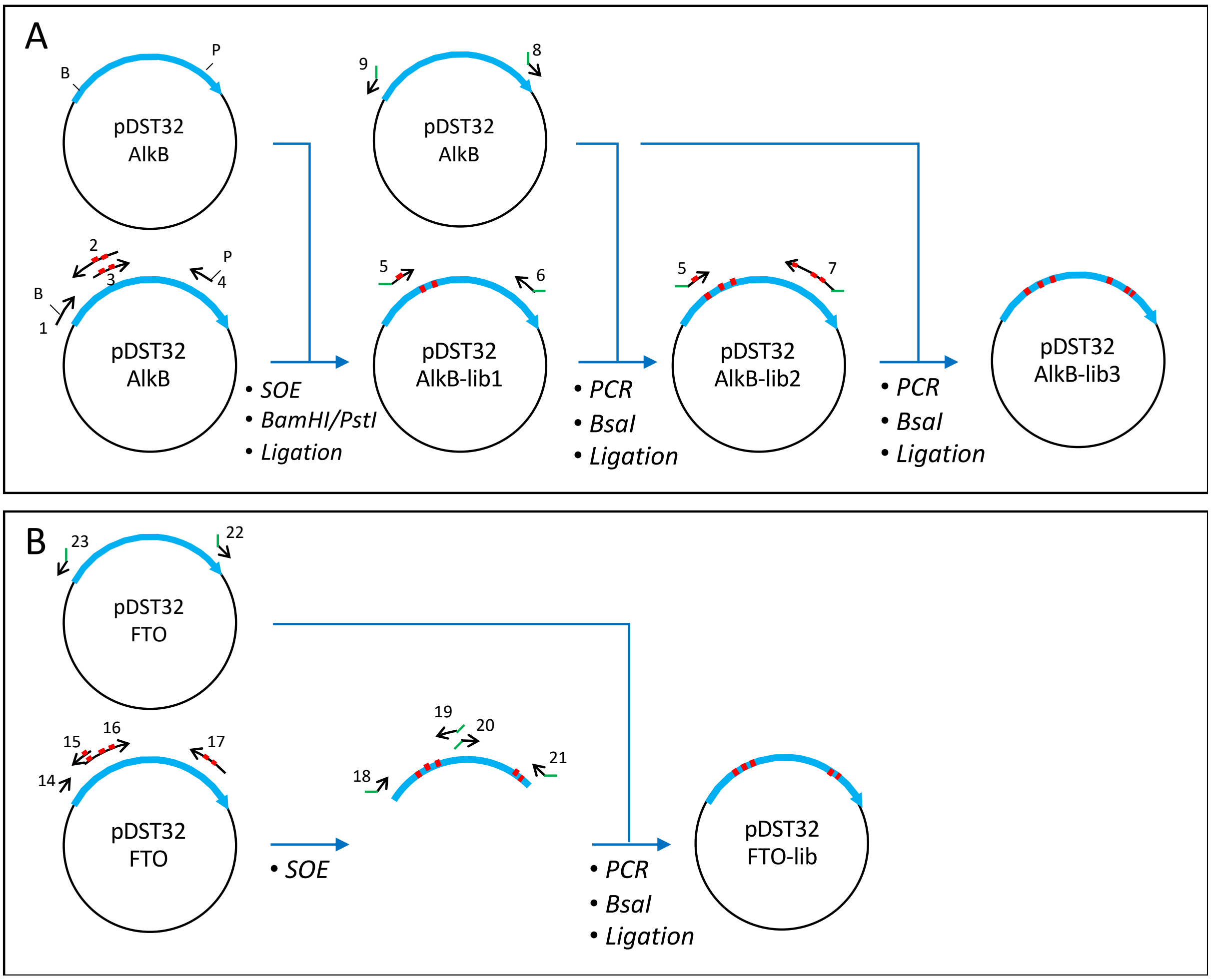

Figure S5. Construction schemes of AlkB (A) and FTO (B) libraries. AlkB and FTO libraries were created in three and two steps, respectively. Mutagenic fragments were generated by PCR using NNK- or TDK-randomized primers (see corresponding numbers in Table S4). Randomized codons are indicated in red. Randomized fragments were fused to ssgIII in phagemid pDST32 using BsaI-assisted restriction and ligation. BsaI-sites are indicated as green primer tails.

A. Construction of wild-type pDST32-AlkB and combinatorial library

A synthetic *alkB* gene fragment (ThermoFisher Scientific) was cloned into phagemid pDST32 using PCR and BamHI/EcoRI restriction. After ligation, phagemids were transformed in *E. coli* and positives clones were verified using colony PCR and Sanger sequencing.

The first library (AlkB-lib1) was generated using overlap extension PCR [2]. First fragments were amplified in two separate PCR reactions, which were purified followed by splicing in a third reaction. Both fragments were mixed and end-filled. After 15 cycles, extra primers containing the restriction sites (primer 1 and 4) were added and the reaction was continued for 15 cycles. The resulting gene fragment was then cloned in pDST32 using BamHI/EcoRI restriction. A combinatorial library was achieved containing all 120 different clones. Next, the first library served as input for the second library (AlkB-lib2). An insert with one extra mutation (primer 5) was amplified by PCR (using primers containing BsaI overhangs (Table S4)), restricted by BsaI (NEB) and ligated. After transformation in *E. coli*, 10^5 clones were obtained, which is less than the 1.6 x 10^7 possible protein-coding combinations. Finally, this second library served as input for the construction of library 3 (AlkB-lib3) in a similar process,

in which 3 extra randomized sites were introduced. After final transformation, 5 x 10^5 TG1 clones were obtained, serving as starting point for phage display. After each transformation step, efficiency of cloning was estimated by the length of PCR products from 30 random clones using a*lkB*-specific primers. Randomization at the target positions was verified using Sanger sequencing.

B. Construction of wild-type pDST32-FTO and combinatorial library

A synthetic gene block of full-length *FTO* sequence (IDT) was cloned into phagemid pDST32 by Gibson assembly [1] using FTO and pDST32-specific overlapping primers (primers 10-13, Table S4). The assembled phagemids were transformed in *E. coli* and positives clones were verified using colony PCR and Sanger sequencing.

First, a 741 bp randomized FTO fragment was generated by overlap extension PCR [2] of two smaller PCR products, using NNK/TDK-randomized primers (primer 14-17) and wild-type pDST32-FTO as template. This randomized FTO fragmented was amplified together with the pDST32-vector PCR fragment using overlapping BsaI-containing primers (primers 18-21). The two PCR products were restricted by BsaI (and DpnI to remove the parental pDST32-FTO phagemid), ligated and transformed into *E. coli*. Notably, phagemids were first transformed into E. cloni (Lucigen) cells due to its better transformation efficiency, obtaining 2.2 x 10^6 independent clones, which is more than the 8 x 10^5 possible protein-coding combinations. Subsequently, phagemids were isolated and retransformed into TG1 strains resulting in a library of 6 x 10^6 clones, which served as starting point for phage display.

After each transformation step, efficiency of cloning was estimated by PCR of 30 random clones using FTO-specific primers. Randomization at the target positions was verified using Sanger sequencing.

[1] D.G. Gibson, L. Young, R.Y. Chuang, J.C. Venter, C.A. Hutchison, H.O. Smith, Enzymatic assembly of DNA molecules up to several hundred kilobases, Nat Methods 6 (2009) 343-345. 10.1038/nmeth.1318.

[2] E.M. Williams, J.N. Copp, D.F. Ackerley, Site-saturation mutagenesis by overlap extension PCR, Methods Mol Biol 1179 (2014) 83-101. 10.1007/978-1-4939-1053-3_6.

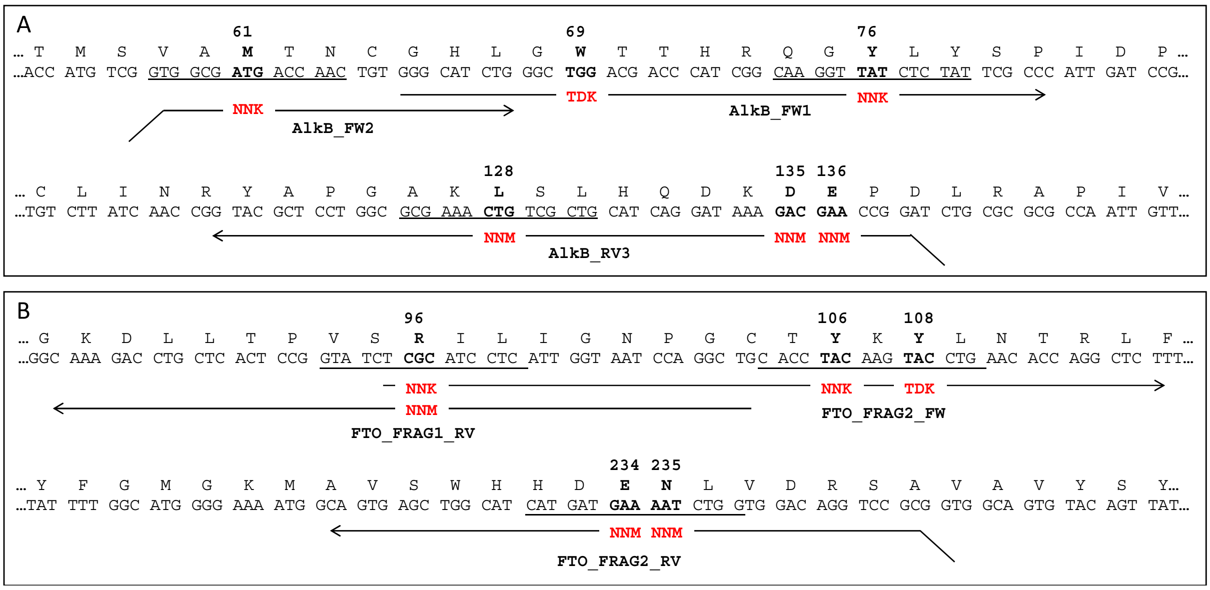

Figure S6. Detailed overview of the targeted regions and randomized codons in *alkB* (A) and *FTO* (B). Target codons are clustered in two distinct areas. Mutagenic primers (see Table S4) are shown with randomized triplets in red. Underlined sequences were used as in silico selection criteria of reads containing the targeted region (see Table S1).

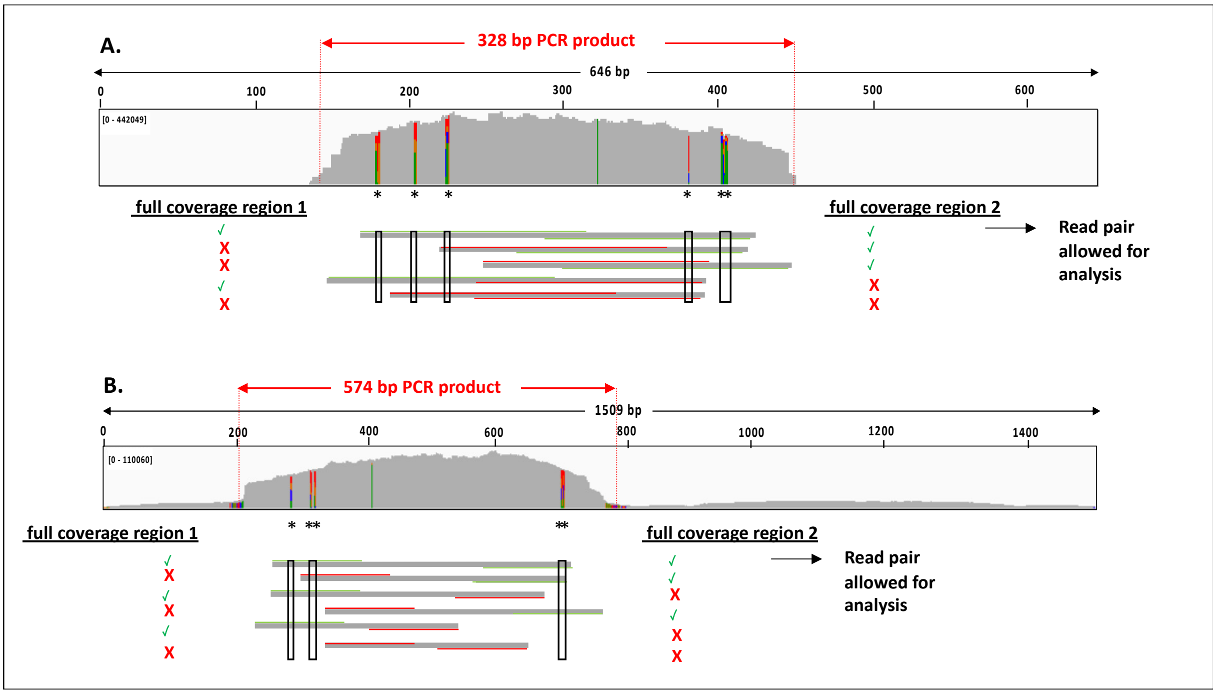

Figure S7. Sequence coverage of the *alkB* (A) and *FTO* (B) gene after deep sequencing of tagmented PCR-amplified target regions (red arrows) of naive library samples. The sequence coverage was calculated after read processing (quality/adapter trimming, pairing) and alignment with the total gene. The image was generated by IGV. Randomized codon positions are indicated with asterisks (*). Green bars represent silent point mutations introduced to remove BamHI and BsaI restrictions sites in *alkB* and *FTO*, respectively. Below the sequence coverage diagram, a cartoon of paired read selection is added. Tagmented fragments (grey lines) vary in length (250-500 bp) and starting position. Illumina sequencing of these fragments yielded 150 bp paired-end reads, either fully (green lines) or incompletely (red lines) covering the randomized regions. Read pairs covering both randomized regions were filtered for further analysis.

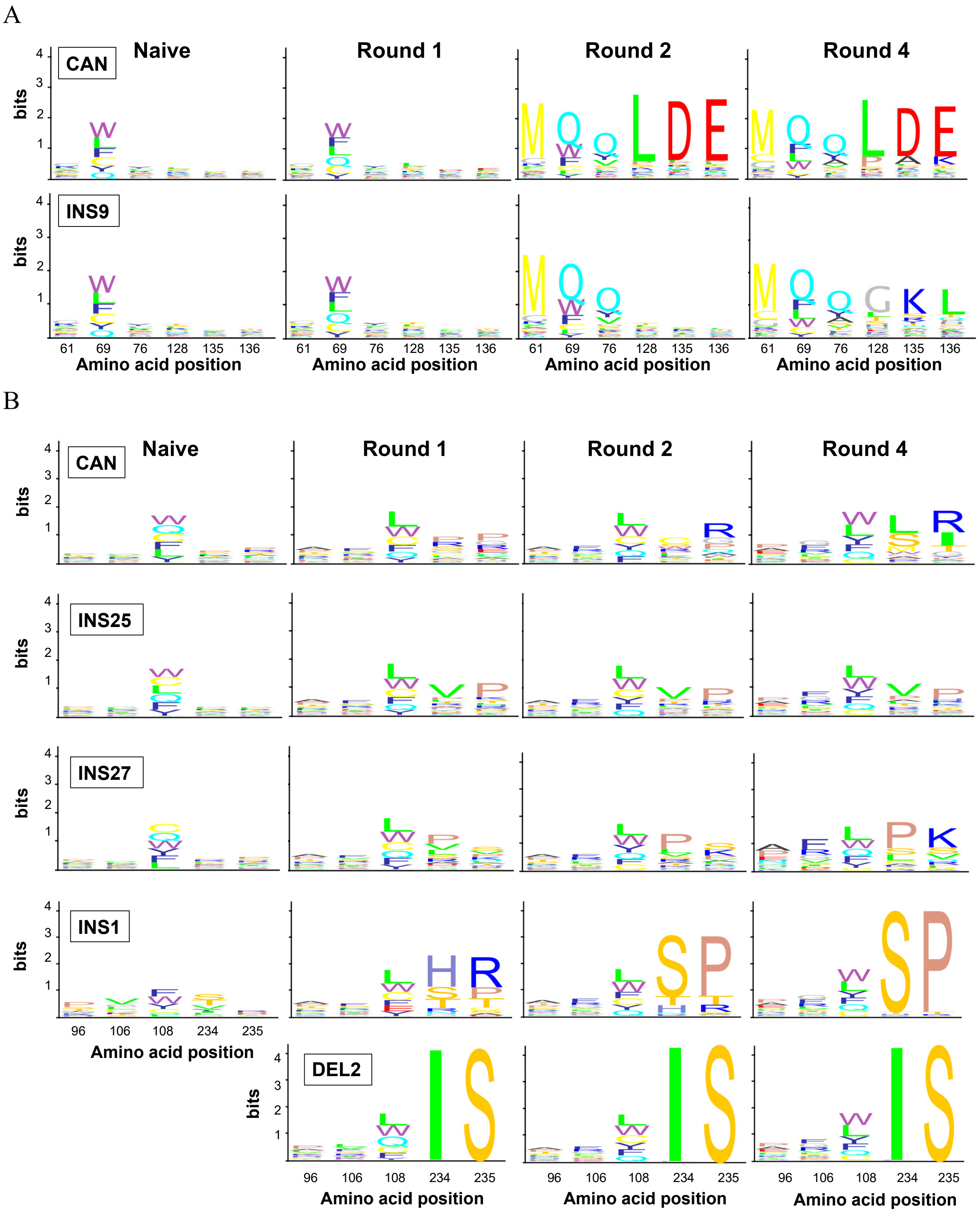

Figure S8. Evolution of amino acids at randomized positions in AlkB and FTO during biopanning as deduced from Illumina DNA sequence reads. A. Sequences logos of canonical (CAN) AlkB clones and insertion variant INS9 at the six randomized positions. B. Sequences logos of canonical (CAN) FTO, insertion mutants INS25, INS27, INS1 and deletion mutant DEL2 at the five randomized positions. Triplet 69 in AlkB and triplet 108 in FTO were TDK-randomized. All other triplets were NNK-randomized. Amber stop codons can be suppressed as Gln, represented as Q.

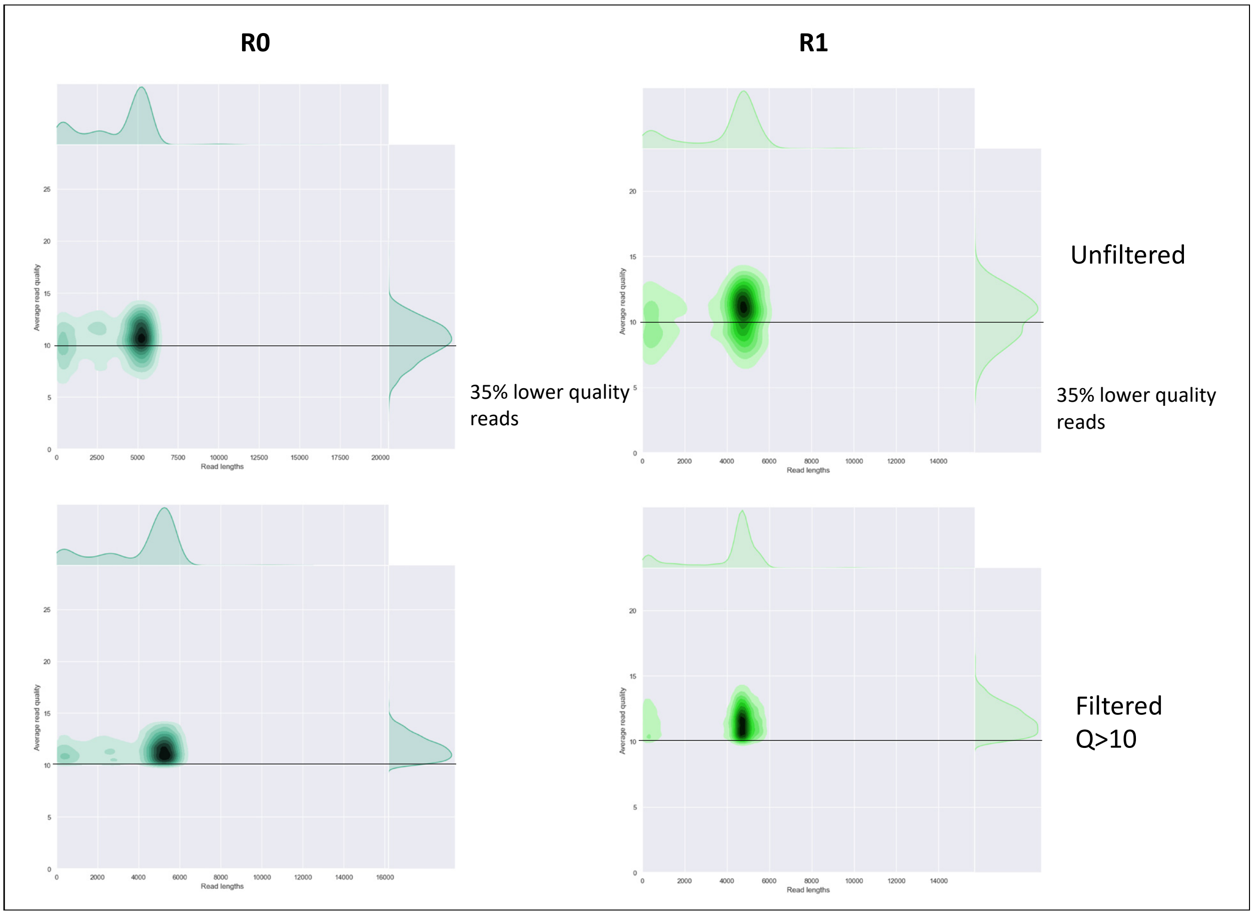

Figure S9. NanoPlot bivariate plot of linearized pDST32-FTO nanopore read lengths against average read quality. Low-quality reads (with Phred score < 10) were filtered out using NanoFilt prior to alignment and sequence coverage calculation.

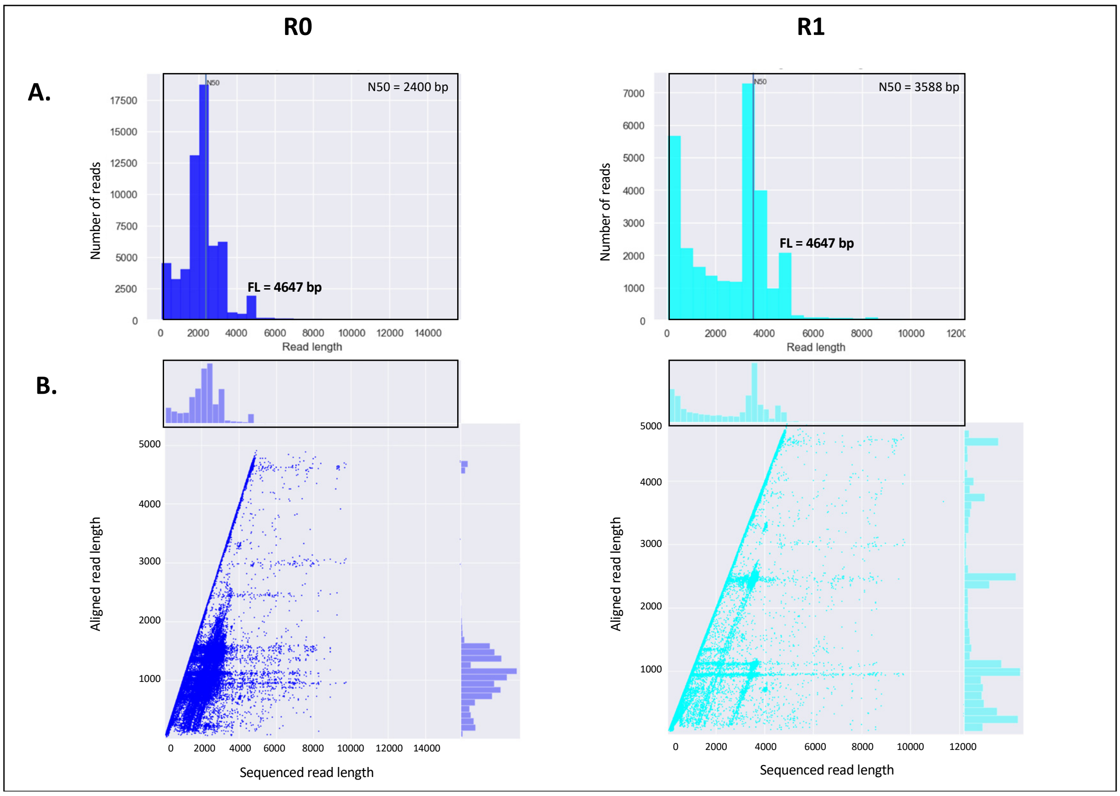

Figure S10. A. NanoPlot histogram of raw nanopore read lengths of naive (R0; blue) and round 1 (R1; cyan) AlkB phagemid libraries and N50 value. B. Bivariate Nanoplot of sequenced read lengths against aligned read lengths.

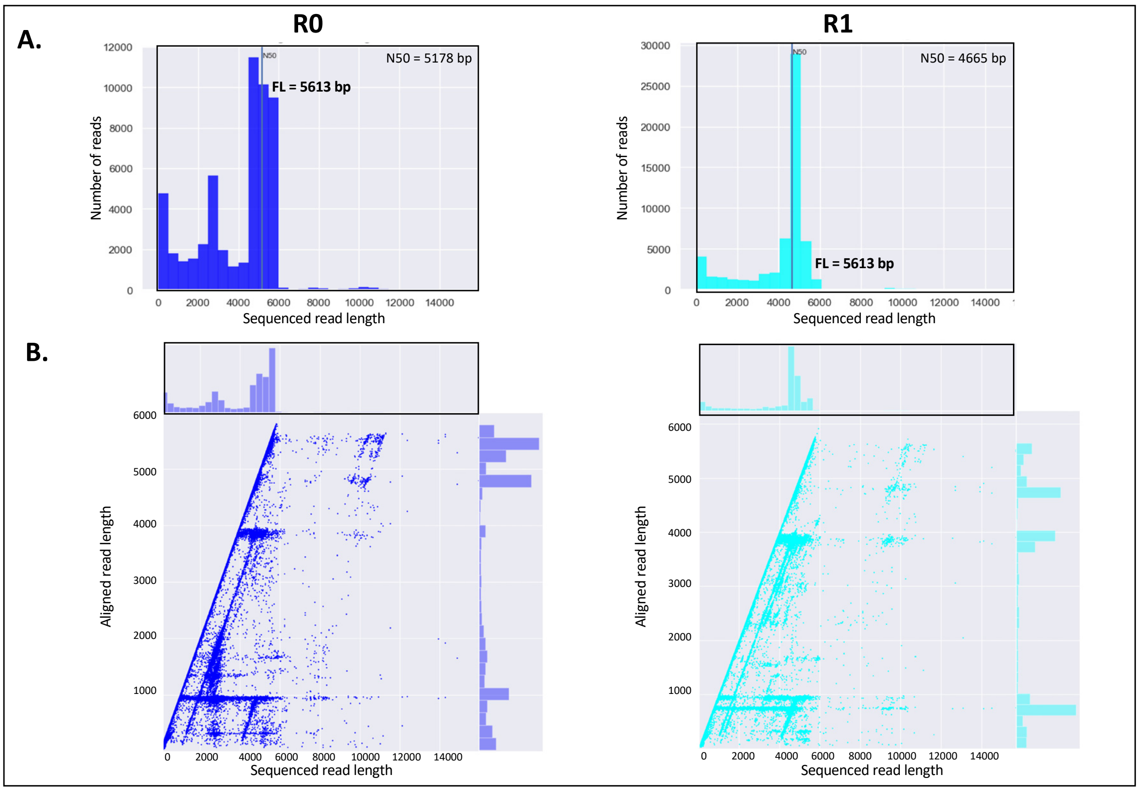

Figure S11. A. NanoPlot histogram of raw nanopore read lengths of naive (R0; blue) and round 1 (R1; cyan) FTO phagemid libraries and N50 value. B. Bivariate Nanoplot of sequenced read lengths against aligned read lengths.

Table S1. Absolute numbers of sequence reads and read pairs in consecutive steps of FASTQ processing.

	Total		After adapter trimming		After quality trimming		After region 1 selection	After region 2 selection	Total paired reads	Paired reads per variant	
**AlkB**	**FW**	**RV**	**FW**	**RV**	**FW**	**RV**				**CAN**	**INS9**
**Naive**	3957987	3957987	3949653	3949653	3824881	3808766	761644	861706	761541	578101	131130
**Round 1**	1208801	1208801	1208200	1208200	1166292	1160798	152168	160860	151953	124990	21489
**Round 2**	1734031	1734031	1733606	1733606	1659914	1642102	140761	154983	140577	121260	5217
**Round 4**	1272582	1272582	1271907	1271907	1211839	1196942	128364	178172	128364	110121	5499

	Total		After adapter trimming		After quality trimming		After region 1 selection	After region 2 selection	Total paired reads	Paired reads per variant				
**FTO**	**FW**	**RV**	**FW**	**RV**	**FW**	**RV**				**CAN**	**INS25**	**INS27**	**INS1**	**DEL2**
**Naive**	1818864	1818864	1818686	1817867	1736177	1723569	115477	162972	115191	77968	1216	167	15	0
**Round 1**	1694336	1694336	1694143	1693343	1617091	1597115	107555	137042	106962	30581	21259	655	810	65
**Round 2**	1674584	1674584	1674413	1673769	1623503	1605470	65062	83117	64156	10713	16190	456	1667	1069
**Round 4**	1491956	1491956	1491824	1491415	1443901	1429849	9977	14132	9453	543	2693	48	761	699

Two data sets containing reads from either forward (FW) or reverse (RV) sequencing primers were adapter- and quality-trimmed. Next, region selection was performed using the presence of targeted sequences (underlined in Figure S6) as filter criteria. Finally, reads were paired and the individual counts for each variant determined.

Table S2. Frequency evolution of most prominent AlkB and FTO amino acid patterns deduced from deep sequencing reads in different selection rounds.

AlkB patterns	Naive (%)		Round 1 (%)		Round 2 (%)		Round 4 (%)	
	CAN	INS9	CAN	INS9	CAN	INS9	CAN	INS9
M*QLDE	0.001	0	0.262	0	27.983	0	23.175	0
CLALDE	0.001	0	0.034	0	0.381	0	5.704	0
M*QPAK	0	0	0.010	0	0.215	0	3.379	0
MWYLDE	0.003	0	0.139	0	3.739	0	3.267	0
M*QGKL	0	0.001	0	0.014	0	2.147	0	19.240

FTO patterns	Naive (%)					Round 1 (%)					Round 2 (%)					Round 4 (%)				
	CAN	INS25	INS27	INS1	DEL2	CAN	INS25	INS27	INS1	DEL2	CAN	INS25	INS27	INS1	DEL2	CAN	INS25	INS27	INS1	DEL2
AFLPP	0.003	0	0	0	/	1.027	0.080	0.611	0	0	0.569	0.117	1.096	0	0	0.184	0	0	0	0
AFLRG	0.001	0	0	0	/	0.755	0.005	0	0	0	0.252	0.006	0	0	0	0	0	0	0	0
AFLLR	0.001	0	0	0	/	0.069	0.056	0	0	0	0.140	0.111	0	0	0	4.236	0.149	0	0	0
EGWLR	0.001	0	0	0	/	0	0	0	0	0	0.047	0.031	0	0	0	3.867	0.037	0	0	0
AFLVP	0	0	0	0	/	0.016	4.445	0	0	0	0.009	4.379	0	0	0	0	3.973	0	0	0
AFLPK	0	0	0	0	/	0	0.005	1.069	0	0	0	0	3.070	0	0	0	0	16.667	0	0
AFLSP	0	0	0	0	/	0.893	0.066	0	2.716	0	0.504	0.093	0.219	13.017	0	0	0	2.083	12.089	0
AFLIS	0	0	0	0	/	0	0	0	0	6.154	0	0	0	0	13.377	0	0	0	0	13.162

The patterns are represented by the sequence of the randomized amino acid residues without the intervening untargeted amino acids. CAN: canonical sequences devoid of observed indels; INS and DEL genotypes (Figure 2). The percentages are calculated by the fraction of each patterns from the total number of amino acid patterns for each variant. A zero-percentage means that the corresponding patterns could not be detected among all patterns for a certain variant. FTO mutant DEL2 was absent in the naive library. Hence, no patterns are presented (indicate as /). The first four FTO patterns were most frequent in the canonical clones; the

next four patterns most frequent in each of the four indel clones. MWYLDE corresponds to the AlkB wild-type pattern. The wild-type FTO pattern (RYYEN) was not detected.

Table S3. Observed *alkB* and *FTO* genotype frequencies and diversities of enriched canonical phenotypes.

**A. AlkB**		**Genotype frequency (%)**			
**Phenotype**	**Genotype**	**Naive**	**Round 1**	**Round 2**	**Round 4**
**M*QLDE**	ATGTAGCAGCTGGACGAA	0.0009	0.3160	32.4039	26.9286
	ATGTAGCAGCTTGACGAA	0	0.0016	0.0107	0.0036
	ATGTAGCAACTGGACGAA	0	0	0.0066	0.0009
	ATGTAGCAGCTGGATGAA	0	0	0.0058	0.0036
	ATGTAACAGCTGGACGAA	0	0	0.0041	0.0027
	ATGTAGCAGCTAGACGAA	0	0	0.0033	0.0018
	ATGTAGCAGCTGGACGAG	0	0	0.0025	0.0073
	ATGTAGCAGCTCGACGAA	0	0	0.0016	0.0200
	ATGTAGCAGCTNGACGAA	0	0	0.0008	0.0009
	ATGTAGCAGTTGGACGAA	0	0.0008	0.0008	0.0027
	ATGTAGCAGTTGGATGAG	0	0	0.0008	0
	**Genotype diversity**	1	3	11	10
**M*QPAK**	ATGTAGCAGCCGGCTAAG	0.0002	0.0120	0.2441	3.923
	ATGTAGCAGCCGGCGAAG	0	0	0.0041	0.0018
	ATGTAACAGCCGGCTAAG	0	0	0	0.0018
	ATGTAGCAGCCTGCTAAG	0	0	0	0.0018
	ATGTAGCAACCGGCTAAG	0	0	0	0.0009
	ATGTAGCAGCCCGCTAAG	0	0	0	0.0009
	ATGTAGCAGCCGGCCAAG	0	0	0	0.0009
	ATGTAGCAGCCGGCTAAA	0	0	0	0.0009
	ATGTAGCAGCCNGCTAAG	0	0	0	0.0009
	**Genotype diversity**	1	1	2	9
**MWYLDE**	ATGTGGTATCTGGACGAA	0.0047	0.168	4.3295	3.794
	ATGTGGTATCTCGACGAA	0.0002	0.0008	0.0016	0.0027
	ATGTGGTATCTAGACGAA	0	0	0.0008	0.0018
	ATGTGGTATCTGGATGAA	0	0	0.0008	0.0018
	ATGTGGTATCTTGACGAA	0	0	0.0008	0.0009
	ATGTGGTATTTGGACGAA	0	0	0.0008	0.0009
	**Genotype diversity**	2	2	6	6
**CLALDE**	TGTTTGGCTCTGGACGAA	0.0014	0.0400	0.4371	6.6200
	TGCTTGGCTCTGGACGAA	0	0.0008	0	0
	TGTTTGGCGCTGGACGAA	0	0	0.0041	0.0054
	TGTTTGGCTCTCGACGAA	0	0	0	0.0064
	TGTTTGGCTCTAGACGAA	0	0	0	0.0018
	TGTTTGGCTTTGGACGAA	0	0	0	0.0018
	TGTTTAGCTCTGGACGAA	0	0	0	0.0009
	TGTTTGGCCCTGGACGAA	0	0	0	0.0009
	TGTTTGGCTCTGGACGAG	0	0	0	0.0009
	TGTTTGGCTCTNGACGAA	0	0	0	0.0009
	**Genotype diversity**	1	2	2	9

**B. FTO**		**Genotype frequency (%)**			
**phenotype**	**genotype**	**naive**	**round 1**	**round 2**	**round 4**
**AFLPP**	GCGTTTTTGCCTCCG	0.0013	0	0	0
	GCTTTTTTGCCTCCG	0.0013	0.0294	0.2894	0
	GCTTTTTTGCCGCCT	0	0.4218	0.0933	0
	GCTTTTTTGCCTCCT	0	0.3597	0.0653	0
	GCTTTTTTGCCGCCG	0	0.2158	0.1213	0.1842
	**Genotype diversity**	2	4	4	1
**AFLRG**	GCTTTTTTGAGGGGT	0	0.7161	0.2520	0
	GCTTTTTTGCGGGGG	0.0013	0.0164	0	0
	GCTTTTTTGCGTGGG	0	0.0098	0	0
	GCTTTTTTGAGGGGC	0	0.0033	0	0
	GCTTTTTTGAGGGGG	0	0.0033	0	0
	GCTTTTTTGCGGGGT	0	0.0033	0	0
	GCTTTTTTGCGTGGT	0	0.0033	0	0
	**Genotype diversity**	1	7	1	0
**AFLLR**	GCTTTTTTGTTGCGG	0.0013	0	0.0187	0
	GCTTTTTTGTTGCGT	0	0.0262	0.0280	4.2357
	GCTTTTTTGCTGAGG	0	0.0196	0.0560	0
	GCTTTTTTGCTGCGG	0	0.0131	0	0
	GCTTTTTTGCTGCGT	0	0.0033	0.0373	0
	GCTTTTTTGCTTCGG	0	0.0033	0	0
	GCTTTTTTGCTTCGT	0	0.0033	0	0
	**Genotype diversity**	1	6	4	1
**EGWLR**	GAGGGTTGGCTTCGG	0.0013	0	0	0
	GAGGGTTGGTTGCGT	0	0	0.0373	3.8674
	GAGGGTTGGCTGAGG	0	0	0.0093	0
	**Genotype diversity**	1	0	2	1

Table S4. Oligonucleotides used in this study.

#	Primer name	Sequence (5' > 3')
1	AlkB_BamHI_FW	TGGT**GGATCC**ATGTTGGATCTGTTTGCCGATGC
2	AlkB_FW1	GGGCATCTGGGCTDKACGACCCATCGGCAAGGTNNKCTCTATTCGCCCAT
3	AlkB_RV1	CGGCGAATAGAGMNNACCTTGCCGATGGGTCGTMHAGC
4	AlkB_PstI_RV	TTCGG**CTGCAG**ATTCTTTTTACCTGCCTGACGG
5	AlkB_FW2	TCC**GGTCTC**TGGCGNNKACCAACTGTGGGCATCTG
6	AlkB_RV2	TCC**GGTCTC**TCCGGTTCGTCTTTATCCTGATGCAGCGACAGTTTCGCGCCAGGAGCGTAG
7	AlkB_RV3	TCC**GGTCTC**TCCGGMNNMNNTTTATCCTGATGCAGCGAMNNTTTCGCGCCAGGAGCGTAG
8	AlkB_VEC_FW	TCC**GGTCTC**ACCGGATCTGCGCGCGCCAATTGTTTCTG
9	AlkB_VEC_RV	TCC**GGTCTC**ACGCCACCGACATGGTATATC
10	FTO_FRAG_GIB_FW	CATCGGCGGACTACAAAGATGGATCCATGGGGAAGCGCA
11	FTO_FRAG_GIB_RV	TCAGAGATCAGCTTCTGCTCGAATTCCTCGAGGGGTTTTGCT
12	FTO_VEC_GIB_FW	CAAAACCCCTCGAGGAATTCGAGCAGAAGCTGATCTCTGAG
13	FTO_VEC_GIB_RV	GTGCGCTTCCCCATGGATCCATCTTTGTAGTCCGCCG
14	FTO_FRAG1_FW	CATCGGCGGACTACAAAGATGGATCCATGGGGAAGCGCA
15	FTO_FRAG1_RV	CCTGGATTACCAATGAGGATMNNAGATACCGGAGTGAGCAGGTCTTTG
16	FTO_FRAG2_FW	TNNKATCCTCATTGGTAATCCAGGCTGCACCNNKAAGTDKCTGAACACCAGGCTCTT
17	FTO_FRAG2_RV	GCGGACCTGTCCACCAGMNNMNNATCATGATGCCAGCTCACTG
18	FTO_BSAI_REG1_FW	ACA**GGTCTC**GGATCCATGGGGAAGCGCAC
19	FTO_BSAI_REG1_RV	ATC**GGTCTC**GTTTCACAAGCAGCGGCTATTTCAGCC
20	FTO_BSAI_REG2_FW	TAG**GGTCTC**TGAAACCTTCCTCAAGCTCAATGAC
21	FTO_BSAI_REG2_RV	GAA**GGTCTCC**GCGGACCTGTCCACCAG
22	FTO_BSAI_VEC_FW	GTA**GGTCTC**TCCGCGGTGGCAGTGTACAG
23	FTO_BSAI_VEC_RV	CAC**GGTCTC**GGATCCATCTTTGTAGTCCGCC
24	FTO_NGS_FW	CTTTCTCACACTGCACAAG
25	FTO_NGS_RV	GTCATCCTCACTTTCCTC
26	AlkB_NGS_FW	CGCCGTTTCGCCAGATGGTCAC
27	AlkB_NGS_RV	ATCGCGGGTAAGCCCAGAGAAACAG
28	pDST32_qPCR_FW	TCATTAAGCATTCTGCCGAC
29	pDST32_qPCR_RV	GTTTTTCGTCTCAGCCAATC
30	M13KO7_qPCR_FW	TACTGATTACGGTGCTATC
31	M13KO7_qPCR_RV	GACTTGAGCCATTTGGGAATT
32	m6A target oligo	(biotinTEG)AAAAAGCGG(m6A)CTCCAGATG
33	m6A competitor	AAAAAGCGGACTCCAGATG
34	m1A target oligo	TAGGTAA(m1A)ACCGTTCCTAGTCCATCTCAGC
35	m1A capture oligo	(biosg-iSp18)GCTGAGATGGACT
36	m1A competitor	TAGGTAAAACCGTTCCTAGTCCATCTCAGC

Randomized triplets are underlined. BamHI, PstI and BsaI sites are in bold. Biotin-TEG indicates biotin coupled via a triethyleneglycol (TEG) spacer; biosg-iSp18 indicates biotin coupled via a C18 internal spacer.
